# Self-Reported Fatigue and Fatigue-Attributed Thoughts of Leaving the Current Job Among Internal Medicine Nurses: An Exploratory Cross-Sectional Study

**DOI:** 10.3390/healthcare14203363

**Published:** 2026-10-09

**Authors:** Biljana Filipović, Milena Gabrić, Danijela Kundrata, Boris Ilić, Franjo Liška, Irena Kovačević

**Affiliations:** 1 Department of Nursing, University of Applied Health Sciences, Mlinarska Cesta 38, 10000 Zagreb, Croatia; boris.ilic@zvu.hr (B.I.); franjo.liska@zvu.hr (F.L.); irena.kovacevic@zvu.hr (I.K.); 2 Faculty of Health Sciences, University of Rijeka, Viktora Cara Emina 5, 51000 Rijeka, Croatia; 3 Clinical Department of Internal Medicine, University Hospital Centre Sestre Milosrdnice, Vinogradska Cesta 29, 10000 Zagreb, Croatia; mgabric15@gmail.com; 4 Department for Ensuring and Improving the Quality of Health Care, General Hospital “Dr. Ivo Pedisic”, Josipa Jurja Strossmayera 59, 44000 Sisak, Croatia; danijela.kundrata@gmail.com

**Keywords:** occupational fatigue, nurses, work schedule, thoughts of leaving, turnover intention, self-report, exploratory survey

## Abstract

Background/Objectives: Work-related fatigue and thoughts of leaving employment are relevant to nursing workforce sustainability, but their measurement requires clear distinctions between symptoms, intentions and observed outcomes. This exploratory study described fatigue, fatigue-attributed thoughts of leaving the current job and perceived occupational consequences among internal medicine nurses. Methods: A cross-sectional survey was conducted in February–March 2025 at a Croatian university hospital. Responses from 145 nurses (57.5% of 252 eligible) were analysed at item level using a study-specific questionnaire, non-parametric tests and logistic regression with sensitivity analyses. Results: Fatigue at least weekly was reported by 129/145 nurses (89.0%; 95% CI 82.8–93.1). Physical and emotional exhaustion several times weekly or daily were reported by 84/145 (57.9%) and 86/145 (59.3%), respectively. Fatigue-attributed thoughts of leaving occurred often or always in 29/145 nurses (20.0%) and at least sometimes in 76/145 (52.4%). Rotating and morning-only nurses showed small or negligible differences in fatigue and exhaustion responses. The adjusted association between rotating work and thoughts of leaving at least sometimes was not statistically significant (OR 2.16, 95% CI 0.997–4.682; *p* = 0.051); inference varied across exploratory model specifications. Frequent self-attributed errors were reported by 8/145 nurses (5.5%). Conclusions: Fatigue was common, but this survey does not establish its causal effects on retention or safety. The findings support further assessment of recovery opportunities and work demands using validated measures and longitudinal designs.

## 1. Introduction

Occupational fatigue encompasses reduced energy and capacity associated with work demands and insufficient recovery; its physical, cognitive and emotional dimensions require careful definition [1,2]. Everyday tiredness, perceived physical exhaustion and perceived emotional exhaustion describe related experiences, but a report of any one does not establish a fatigue syndrome. Emotional exhaustion is also a component of burnout, whereas burnout includes broader features such as cynicism or depersonalisation and reduced professional efficacy. General occupational stress concerns responses to work demands and is not interchangeable with either fatigue or burnout [3]. Accordingly, this study uses the wording of individual questions and does not interpret them as diagnostic measures of burnout or stress.

Validated instruments distinguish dimensions that a single item cannot represent. The Occupational Fatigue Exhaustion/Recovery scale (OFER) separates acute fatigue, chronic fatigue and recovery between shifts [2]. Recent psychometric research on the Nurse Turnover Intention Scale has evaluated content, structural and criterion-related validity as well as temporal reliability in nurses [4]. In contrast, a question about considering departure specifically because of fatigue captures a narrow, self-attributed experience. It is neither equivalent to a validated multidimensional turnover intention measure nor a measure of subsequent staff turnover.

International findings illustrate both the relevance of these experiences and the importance of measurement differences. Among 2140 nurses from 186 Chinese hospitals, an OFER-based analysis identified moderate-fatigue/moderate-recovery and high-fatigue/low-recovery profiles in 48.8% and 32.6%, respectively [5]. In a European survey of 1351 nurses, 8.4% expressed an intention to leave their hospital and 13.6% to leave nursing [6]. These estimates use different instruments, targets and response definitions; they are contextual evidence rather than prevalence benchmarks for a fatigue-attributed single item.

The Croatian context is relevant to workforce sustainability without implying that local working conditions are uniformly worse than elsewhere. The 2025 Country Health Profile reports 7.7 nurses per 1000 population in Croatia in 2023, compared with an EU average of 8.5, with all Croatian nursing categories included [7]. A regional study including Croatian nursing staff found lower job-satisfaction scores in Central and Eastern Europe than in the comparison regions [8]. Croatian research has also documented poorer objectively assessed sleep among rotating-shift nurses [9]. National workforce indicators and regional findings provide context; they do not establish staffing adequacy or individual exposures in the present department.

Internal medicine provides a useful setting for examining fatigue across a broad range of nursing activities, including inpatient medical care, intensive care, dialysis and day-hospital services. The present study does not assume that internal medicine nurses experience more fatigue than nurses in other specialties. Its focus is the coexistence of fatigue, perceived consequences and fatigue-attributed thoughts of leaving within one clinical department, and whether a broad schedule classification distinguishes these experiences.

The Job Demands-Resources (JD-R) framework provides a conceptual basis for considering how work demands and recovery resources may relate to staff experiences [10]. Long shifts and perceived insufficient breaks can be considered potential demands or constraints on recovery. However, the questionnaire does not operationalise the full JD-R model, and the study does not test its pathways, mediation or buffering effects. The framework is used to organise interpretation and generate questions for subsequent research.

The primary aim was to describe the frequency of self-reported fatigue and fatigue-attributed thoughts of leaving the current job. Secondary aims were to describe perceived occupational consequences and explore differences by work schedule, education and experience. Three propositions guided the analyses: (H1) more than half of respondents report fatigue at least weekly; (H2) fatigue is reported more frequently by nurses on rotating 12 h schedules than by morning-only nurses; and (H3) nurses with longer experience report fatigue-attributed thoughts of leaving more frequently. The study contributes an item-level account that separates fatigue from self-attributed consequences and different targets of departure; examines whether broad schedule categories distinguish these experiences; and evaluates how the interpretation of thoughts of leaving depends on the response threshold. These contributions are exploratory rather than tests of causal mechanisms.

## 2. Materials and Methods

### 2.1. Study Design and Setting

A cross-sectional survey was conducted at the Clinical Department of Internal Medicine, University Hospital Centre Sestre milosrdnice, Zagreb, Croatia, between February and March 2025. Eligible services included endocrinology, diabetes and metabolic diseases; gastroenterology and hepatology; intensive medicine; dialysis; nephrology and arterial hypertension; pulmonology; clinical immunology and rheumatology; haematology; endoscopy and day-hospital services; clinical pharmacology; and infectious diseases. The 12/24/12/48 roster comprises a 12 h day shift, 24 h off, a 12 h night shift and 48 h off. Both day and night shifts in this roster last 12 h. The questionnaire did not record the clinical unit, to protect anonymity, or individual nurse-to-patient ratios, monthly working hours or night shift counts. Reporting follows the STROBE statement [11]; Supplementary File S2 contains the checklist.

### 2.2. Participants and Sample Size

All nurses employed in the department and present during the collection period were eligible, irrespective of length of service; newly employed nurses were included. Absence throughout the collection period, such as long-term leave, was the only exclusion criterion. Of 280 employed nurses, 28 were absent throughout the period, leaving 252 eligible nurses. A census of this eligible population was attempted, so no a priori sample-size calculation was performed. A total of 145 questionnaires were returned (145/252, 57.5%), and none was excluded.

A member of the research team employed in the department (M.G.) invited nurses to participate and distributed paper questionnaires to all 252 eligible nurses. Questionnaires were completed during working hours and returned to a sealed collection box. Participation was voluntary. Signed consent forms were collected separately from questionnaires to preserve anonymity. Returned questionnaires with isolated missing responses were retained, and analyses used the available data for each variable. Reasons for non-participation and characteristics of non-responders were not recorded; the response process therefore did not support a comparison between responders and non-responders.

### 2.3. Instrument

A study-specific questionnaire was developed in Croatian to explore selected experiences of fatigue, perceived occupational consequences and thoughts of leaving. Its conceptual content drew on the literature concerning fatigue and exhaustion [1,3], sleep loss and work schedules [12,13,14], fatigue risk management and performance [15], and staff turnover and intentions to leave [6,16]. These sources informed the selection of topics; the questionnaire was not an adaptation of a validated scale. The English translation was prepared for reporting; the questionnaire was developed and used in Croatian, so no formal cross-language adaptation or back-translation validation procedure was undertaken before data collection.

The draft was reviewed independently for relevance, clarity and comprehensibility by six members of the author team: four academic nursing researchers (B.F., B.I., F.L. and I.K.) and two clinical nursing professionals with hospital-practice and healthcare-quality experience (M.G. and D.K.). This was an internal qualitative review rather than an independent external validation panel. No pilot study, cognitive interviews, item-level or scale-level content validity indices, test–retest assessment or construct-validation study was conducted. The instrument is therefore described as a study-specific, item-based questionnaire with limited validity evidence.

The present analyses use 21 variables: five sociodemographic and work-related variables, eight items on fatigue and work experiences (items 6–13), and eight items on perceived consequences and thoughts of leaving (items 14–21). Each item was analysed separately; no total or subscale scores were calculated. Because the sections cover heterogeneous constructs rather than established unidimensional scales, Cronbach’s alpha is not used to support their reliability or validity. Supplementary File S1 provides the submitted English questionnaire and informed consent text, with all response options, together with a Croatian translation prepared for this revision.

For items 6, 12 and 13, codes 1–5 correspond to every day, several times per week, once per week, rarely and never. For items 7–9, 11, 14–16 and 18–21, codes 1–5 correspond to always, often, sometimes, rarely and never. Item 10 ranges from very satisfied (1) to very dissatisfied (5), and item 17 from very negative (1) to very positive (5). Lower values therefore indicate more frequent experiences, greater satisfaction or a more negative perceived communication effect, depending on the item. Appendix A summarises the analysed items.

Item 8 asks how often respondents consider leaving their current job because of fatigue. It is treated throughout as an exploratory, self-reported indicator of fatigue-attributed thoughts of leaving, not as a validated measure of the multidimensional construct of turnover intention. Item 19 refers to considering another position within the same institution because of fatigue. Items 20 and 21 concern another institution and departure from the profession, respectively, without attributing these thoughts to fatigue. None measures an actual departure.

### 2.4. Statistical Analysis

Age and years of experience recorded in the analytical dataset are summarised as means, standard deviations (SDs), and ranges. Ordinal items are reported as medians and interquartile ranges (IQRs; Q3 − Q1), and categorical responses as n/N and percentages. Quartiles use interpolation at positions (n + 1)p. Wilson 95% confidence intervals (CIs) accompany key proportions. Isolated missing data were handled by available-case analysis without imputation.

For H1, fatigue at least weekly means every day, several times per week or once per week on item 6 (codes 1–3); its proportion was compared with 0.50 using a one-sample binomial test. The more frequent category reported for items 6, 12 and 13 comprises every day or several times per week (codes 1–2). For the remaining frequency items, frequent means always or often (codes 1–2), and at least sometimes includes always, often and sometimes (codes 1–3). The principal binary definition for item 8 contrasts thoughts occurring at least sometimes with rarely or never. This descriptive threshold distinguishes recurring or occasional thoughts from infrequent or absent thoughts; it is not a validated clinical cut-off. The more stringent frequent-thoughts definition was examined in a sensitivity analysis. Schedule satisfaction combines very satisfied and satisfied, and a negative communication effect combines very negative and negative. Each table identifies its numerator, denominator and threshold.

The main schedule comparison contrasted rotating 12/24/12/48 work with morning-only work, using two-sided Mann–Whitney U tests for items 6, 12, 13 and 9. This comparison separates the rotating day/night pattern from a uniform daytime pattern; the seven nurses with combined morning/afternoon work were excluded from this particular contrast but were retained in descriptive and other eligible analyses. These comparisons are exploratory, and their four *p*-values were adjusted together using Holm’s method before rounding. Absolute rank-biserial correlations describe the magnitude of differences. Supplementary analyses compare all three recorded schedules or combine both daytime schedules. Categorical associations use Fisher–Freeman–Halton exact tests for 3 × 2 tables and Fisher’s exact test for 2 × 2 tables; Cramér’s V describes association strength. For H3, the original descriptive split at 15 years of experience was retained solely for an exploratory group comparison; it is not a clinical or externally validated threshold. Experience was also retained continuously in regression.

An exploratory binary logistic regression estimated associations with item 8 responses occurring at least sometimes. All specified covariates were entered simultaneously, without significance-based variable selection: rotating work (1) versus either daytime schedule (0), female (1) versus male (0), years of experience per year, and two education indicators comparing bachelor’s and master’s preparation with secondary nursing education. Age was excluded because it was strongly correlated with experience (Pearson r = 0.973). The model included 142 nurses with complete covariate data: 76 events and 66 non-events. One nurse with missing sex, one who preferred not to disclose sex and one with missing experience were excluded. The undisclosed response was not assigned to either sex category, and a separate category containing one respondent was not estimated.

Model assessment included convergence, the likelihood-ratio test against an intercept-only model, Akaike’s information criterion (AIC), Nagelkerke’s pseudo-R^2^, the Brier score and variance inflation factors (VIFs). Sensitivity analyses conducted during revision used the stricter item 8 threshold; omitted sex to retain nurses with missing or undisclosed sex, with a corresponding model in the original complete-case sample; and added a centred quadratic experience term to examine sensitivity to the linearity assumption. These analyses assess robustness and do not replace the main model on the basis of statistical significance. A multivariable model for weekly fatigue was not fitted because only 16 respondents were in the less frequent category. Two-sided *p*-values and effect estimates with CIs are interpreted together; the nominal significance threshold was 0.05. Original analyses used Microsoft Excel (Microsoft 365; Microsoft Corp., Redmond, WA, USA) and IBM SPSS Statistics 29.0 (IBM Corp., Armonk, NY, USA), and numerical verification and sensitivity analyses used Python 3.12 (Python Software Foundation, Wilmington, DE, USA) with SciPy 1.17.0. Supplementary File S3 provides response distributions, subgroup denominators and additional model results.

### 2.5. Ethical Considerations

The study was conducted in accordance with the Declaration of Helsinki and approved on 11 February 2025 by the Ethics Committee of the University Hospital Centre Sestre milosrdnice, Zagreb, Croatia, the institution in which recruitment and data collection took place (Class: 003-06/25-03/007; Approval No. 251-29-11/3-25-23). Written informed consent was obtained from all participants. Signed consent forms were stored separately from the anonymous questionnaires.

## 3. Results

### 3.1. Sample Characteristics

The sample comprised 145 nurses. The mean age recorded in the dataset was 39.3 (SD 13.1) years, with a range of 20–65 years. Mean experience was 18.4 (SD 13.6) years among 144 nurses, with a range of 0–45 years. Women accounted for 128/145 respondents (88.3%); one nurse preferred not to disclose sex and one left it blank. Secondary nursing education was reported by 70/145 (48.3%), a bachelor’s degree by 63/145 (43.4%) and a master’s degree by 12/145 (8.3%). Rotating work was reported by 81/145 (55.9%), morning-only work by 57/145 (39.3%) and combined morning/afternoon work by 7/145 (4.8%) (Table 1).

### 3.2. Frequency and Self-Assessment of Fatigue

Fatigue at least weekly was reported by 129/145 nurses (89.0%; 95% CI 82.8–93.1), exceeding the 50% reference proportion (*p* < 0.001) and supporting H1 descriptively. Fatigue several times per week or daily was reported by 103/145 (71.0%), physical exhaustion by 84/145 (57.9%) and emotional exhaustion by 86/145 (59.3%). Fatigue often or always affected perceived ability to work for 58/145 nurses (40.0%), while 63/145 (43.4%) frequently attributed fatigue to insufficient breaks. Most respondents were satisfied or very satisfied with their schedule (106/145, 73.1%). Thoughts of leaving the current job because of fatigue occurred often or always in 29/145 (20.0%) (Table 2).

### 3.3. Fatigue by Work Schedule, Education, and Length of Service

Rotating and morning-only nurses did not differ significantly in item 6 fatigue, physical exhaustion or emotional exhaustion after Holm adjustment; the absolute rank-biserial correlations were 0.11, 0.07 and 0.06, respectively. These estimates represent small or negligible observed differences rather than evidence of equivalence. The attribution of fatigue to long shifts differed (unadjusted *p* = 0.003; Holm-adjusted *p* = 0.011; |r| = 0.29). Identical group medians indicate that this finding concerns the distribution of responses rather than a difference in medians (Table 3). H2 was not supported in the main exploratory contrast.

Weekly fatigue was not associated with education in the exploratory categorical analysis (exact *p* = 0.214; Cramér’s V = 0.13). It was reported by 76/81 rotating nurses (93.8%), 49/57 morning-only nurses (86.0%) and 4/7 morning/afternoon nurses (57.1%). The three-category comparison was significant (exact *p* = 0.012; V = 0.26) but depended on a subgroup of only seven nurses. Combining the two daytime schedules gave 53/64 nurses (82.8%) reporting weekly fatigue; the rotating-versus-daytime comparison was not significant (Fisher’s exact *p* = 0.059; unadjusted OR 3.15). These results do not support a stable schedule association across group definitions. Full subgroup counts appear in Supplementary File S3.

For the exploratory experience comparison, 68 nurses had fewer than 15 years and 76 had at least 15 years of experience; one value was missing. Item 8 responses did not differ significantly (medians 3.5 and 3.0; *p* = 0.737; |r| = 0.03). Schedule satisfaction (*p* = 0.326), perceived contribution of insufficient breaks (*p* = 0.451) and item 6 fatigue (*p* = 0.650) also did not differ significantly. H3 was not supported. Thoughts of leaving at least sometimes were reported by 76/145 nurses (52.4%), compared with 69/145 (47.6%) reporting rarely or never. Their frequency under this binary definition was not associated with education (exact *p* = 0.655; V = 0.08) or the three recorded schedules (exact *p* = 0.143; V = 0.17).

### 3.4. Perceived Consequences of Fatigue

Fatigue was perceived to affect patient safety often or always by 37/145 nurses (25.5%). Self-attributed work errors occurred often or always for 8/145 nurses (5.5%), at least sometimes for 46/145 (31.7%), and rarely or never for 99/145 (68.3%). Reduced productivity at least sometimes was reported by 102/145 (70.3%), while 96/145 (66.2%) described a neutral effect on communication. Frequent thoughts of transfer within the same institution because of fatigue were reported by 23/145 (15.9%). Frequent thoughts of moving to another institution occurred in 17/144 (11.8%), and of leaving nursing in 33/145 (22.8%); these latter items did not refer to fatigue (Table 4). All measures describe respondents’ perceptions, not observed incidents or actual departures.

### 3.5. Exploratory Multivariable Associations

The main model included 142 nurses, comprising 76 events (53.5%) and 66 non-events. Rotating work showed a non-significant tendency towards higher odds of fatigue-attributed thoughts of leaving at least sometimes (adjusted OR 2.16, 95% CI 0.997–4.682; *p* = 0.051). No other covariate was statistically significant (Table 5). The model converged, with a likelihood-ratio χ^2^ of 5.38 (5 degrees of freedom, *p* = 0.371), AIC of 202.77, Nagelkerke’s R^2^ of 0.050 and Brier score of 0.240. VIFs ranged from 1.08 to 1.30.

With the stricter often/always outcome, 29/142 nurses were events and the adjusted rotating-work OR was 1.55 (95% CI 0.61–3.96; *p* = 0.360). Omitting sex and retaining the two nurses with missing or undisclosed sex gave *n* = 144 and an OR of 2.23 (95% CI 1.04–4.77; *p* = 0.040); the same covariate specification restricted to the original 142 nurses gave an OR of 2.12 (95% CI 0.98–4.58; *p* = 0.055). A quadratic experience term improved fit relative to the linear model in an exploratory likelihood-ratio comparison (*p* = 0.049); the corresponding rotating-work OR was 2.35 (95% CI 1.07–5.18; *p* = 0.034). The estimates are imprecise and statistical significance changes with model specification. These sensitivity analyses therefore do not establish a robust association (Supplementary File S3).

## 4. Discussion

This exploratory survey documents frequent fatigue alongside less frequent fatigue-attributed thoughts of leaving and self-attributed work errors. These observations concern different experiences and response thresholds, rather than a single continuum of fatigue severity. The principal contribution is to show why item content, attribution and threshold selection matter when interpreting apparently discordant occupational-health findings.

### 4.1. Interpreting the Small Schedule Differences

The absence of statistically significant differences in fatigue and exhaustion responses does not demonstrate that rotating and morning-only schedules are equivalent. The observed differences were small, while a broad roster label omits variation in workload, night shift frequency, recovery and clinical setting. The Chinese OFER study linked fatigue profiles to night shift frequency and work-related effort [5]; a prospective study of nurses working consecutive nights also examined sleep and fatigue at a more detailed temporal level [14]. These findings support more granular exposure measurement than was available here.

Viewed through JD-R, the present pattern suggests a question for future research: do demands and recovery constraints shared across schedules help explain frequent fatigue in both groups? The frequent attribution of fatigue to insufficient breaks is compatible with this interpretation but does not test it. Likewise, high schedule satisfaction can coexist with fatigue because satisfaction with a roster and recovery adequacy are distinct experiences. JD-R remains an interpretive framework; no measured resource-buffering or mediation mechanism is inferred [10].

### 4.2. Thoughts of Leaving and Sensitivity to Measurement

Item 8 distinguishes thoughts attributed to fatigue from broader intentions to change institutions or leave nursing. Its frequent-response prevalence of 20.0% and at-least-sometimes prevalence of 52.4% answer different descriptive questions. The former captures more recurrent thoughts, whereas the latter includes occasional consideration. Neither threshold identifies a clinical condition or predicts an actual departure. Comparisons with studies using defined future intentions, such as the European hospital survey, are therefore conceptual rather than direct prevalence comparisons [6].

The main adjusted estimate for rotating work was imprecise and did not meet the nominal significance threshold. The weaker estimate under the stricter outcome definition and the crossing of *p* = 0.05 under other model specifications reinforce the fragility of inference. These results provide no basis for concluding that schedule organisation is a demonstrated cause of thoughts of leaving or that changing schedules will improve retention. They identify measurement and modelling choices that should be addressed prospectively with a larger sample and validated instruments.

### 4.3. Frequent Fatigue and Less Frequent Self-Attributed Errors

Frequent fatigue and less frequent self-attributed errors should not be interpreted as evidence that fatigue is harmless or that the department has a low actual error rate. The fatigue item asks about a personal experience, while the error item additionally requires recognition of an event and attribution of that event to fatigue. These different cognitive demands, response thresholds and potential social-desirability effects can contribute to different response distributions. Recall and attribution bias are plausible explanations, not findings established by this survey. Earlier nursing research linked extended working hours with reported errors [13], but its event recording and exposure definitions differ from the present question. Incident records, prospective fatigue measurements and validated safety-culture measures would be needed to examine the relationship more directly.

### 4.4. Practical Implications

The findings support assessing recovery opportunities across all recorded schedules rather than focusing only on rotating workers. Since 63/145 nurses frequently attributed fatigue to insufficient breaks, a practical next step is to assess whether planned breaks can actually be taken and how this varies with patient dependency and staffing. Frequent thoughts of leaving also justify confidential discussions about retention concerns that distinguish fatigue-attributed thoughts from other reasons for departure. These are priorities for organisational assessment, not interventions whose effectiveness was demonstrated by this study. Broader fatigue risk-management approaches may guide such work [15], but staffing changes, roster redesign and retention initiatives should be evaluated prospectively.

### 4.5. Limitations

The single-centre, cross-sectional design limits generalisability and precludes temporal or causal inference. The questionnaire contains purpose-designed items with limited validity evidence. Qualitative review by the author team does not establish content or construct validity, and no pilot testing, cognitive interviews, quantitative content validity indices, test–retest reliability or convergent/criterion validity assessments were performed. In particular, item 8 is a single exploratory indicator, not a validated turnover intention scale. The questions do not specify a uniform retrospective reference period. Self-report, recall, attribution and social-desirability biases may affect all responses, particularly reported errors. Formal incidents and safety culture were not assessed.

The response rate of 57.5% creates a material risk of non-response bias. Reasons for non-participation and characteristics of non-responders were not collected, so its direction and magnitude cannot be assessed. Participation may relate to fatigue or willingness to discuss occupational concerns, but the study does not establish such a relationship. Completion during working hours and invitation by a departmental colleague may also influence participation or responses despite voluntary participation and separate consent collection. Clinical unit, staffing ratios, working hours and night shift frequency were not measured, limiting adjustment for workload and case mix and preventing estimation of the proportion working in intensive care.

The small morning/afternoon and master’s-prepared groups limit subgroup inference. Dichotomisation loses ordinal information, and no threshold for item 8 has established clinical validity. The sensitivity analyses demonstrate uncertainty associated with outcome definition, case inclusion and the functional form of experience. The stricter outcome model has only 29 events for five predictor coefficients and should be interpreted particularly cautiously. Multiple exploratory comparisons, absence of an a priori sample-size calculation and broad CIs further limit inference. Non-significant results do not establish absence of an association or equivalence between groups.

## 5. Conclusions

Self-reported fatigue was common among nurses in this internal medicine department, while frequent fatigue-attributed thoughts of leaving and self-attributed errors were less commonly reported. The main analyses did not establish clear associations with broad schedule categories, education or experience; exploratory sensitivity analyses showed that the interpretation of rotating work and thoughts of leaving depends on analytical choices. The findings warrant assessment of recovery opportunities and further research, but do not demonstrate that fatigue causes departures, errors or reduced patient safety. Future multicentre, longitudinal studies should culturally adapt and evaluate established multidimensional occupational fatigue and turnover intention instruments for Croatian-speaking nurses, including cognitive testing, content and construct validity, and test–retest reliability [2,4]. Such studies should measure staffing, workload and recovery alongside prospective occupational outcomes.

## Figures and Tables

**Table 1:** Sociodemographic characteristics and work schedules (N = 145).

Characteristic	Value
Age, years; mean (SD); range; valid *n*	39.3 (13.1); 20–65; 145
Healthcare experience, years; mean (SD); range; valid *n*	18.4 (13.6); 0–45; 144
Female	128/145 (88.3%)
Male	15/145 (10.3%)
Sex: prefer not to disclose	1/145 (0.7%)
Sex: missing	1/145 (0.7%)
Secondary nursing education	70/145 (48.3%)
Bachelor’s degree	63/145 (43.4%)
Master’s degree	12/145 (8.3%)
Rotating 12/24/12/48	81/145 (55.9%)
Morning only	57/145 (39.3%)
Combined morning/afternoon	7/145 (4.8%)

**Table 2:** Responses to fatigue-related items and schedule satisfaction (items 6–13).

Item and Threshold	n/N (%) [95% CI]	Median (IQR)
6. Fatigue at work (daily/several times weekly)	103/145 (71.0) [63.2–77.8]	2.0 (1.0)
7. Fatigue affects ability to work (always/often)	58/145 (40.0) [32.4–48.1]	3.0 (1.0)
8. Thoughts of leaving because of fatigue (always/often)	29/145 (20.0) [14.3–27.2]	3.0 (1.0)
9. Fatigue attributed to long shifts (always/often)	55/145 (37.9) [30.4–46.0]	3.0 (2.0)
10. Satisfied with schedule (very satisfied/satisfied)	106/145 (73.1) [65.4–79.7]	2.0 (1.0)
11. Fatigue attributed to insufficient breaks (always/often)	63/145 (43.4) [35.7–51.6]	3.0 (1.0)
12. Physical exhaustion at work (daily/several times weekly)	84/145 (57.9) [49.8–65.7]	2.0 (1.0)
13. Emotional exhaustion because of work (daily/several times weekly)	86/145 (59.3) [51.2–67.0]	2.0 (1.5)

Each row specifies its response threshold. Proportions are n/N (%) with Wilson 95% CIs. For frequency items, lower values indicate more frequent experiences; for item 10, lower values indicate greater satisfaction. IQR = Q3 − Q1. The weekly definition for item 6 includes once per week in addition to the two categories shown in its row; this gives 129/145 (89.0%). For item 8, the additional at-least-sometimes definition gives 76/145 (52.4%).

**Table 3:** Responses to fatigue-related items among rotating and morning-only nurses.

Item	Rotating *n* = 81 Median (IQR)	Morning *n* = 57 Median (IQR)	*p*	Holm *p*	|r|
6. Fatigue at work	2.0 (1.5)	2.0 (1.0)	0.243	0.728	0.11
12. Physical exhaustion at work	2.0 (1.0)	2.0 (2.0)	0.466	0.933	0.07
13. Emotional exhaustion because of work	2.0 (1.0)	2.0 (1.5)	0.566	0.933	0.06
9. Fatigue attributed to long shifts	3.0 (1.0)	3.0 (2.0)	0.003	0.011	0.29

Group sizes are 81 rotating and 57 morning-only nurses; all four items are complete in these groups. The seven morning/afternoon nurses are excluded only from this contrast. IQR = Q3 − Q1. *p*: two-sided Mann–Whitney U test with tie and continuity corrections. Holm *p*: adjustment across all four unrounded *p*-values. |r|: absolute rank-biserial correlation. Item 9 assesses respondents’ attribution to long shifts, not an objectively measured exposure–outcome relationship.

**Table 4:** Perceived consequences of fatigue (items 14–21).

Item and Threshold	n/N (%) [95% CI]	Median (IQR)
14. Fatigue affects patient safety (always/often)	37/145 (25.5) [19.1–33.2]	3.0 (2.0)
15. Fatigue affects decision-making (always/often)	28/145 (19.3) [13.7–26.5]	3.0 (1.0)
16. Errors attributed to fatigue (always/often)	8/145 (5.5) [2.8–10.5]	4.0 (1.0)
17. Negative effect on communication (very negative/negative)	44/145 (30.3) [23.4–38.3]	3.0 (1.0)
18. Fatigue reduces productivity (always/often)	29/145 (20.0) [14.3–27.2]	3.0 (1.0)
19. Thoughts of internal transfer because of fatigue (always/often)	23/145 (15.9) [10.8–22.7]	4.0 (2.0)
20. Thoughts of moving to another institution (always/often)	17/144 (11.8) [7.5–18.1]	4.0 (2.0)
21. Thoughts of leaving nursing (always/often)	33/145 (22.8) [16.7–30.2]	4.0 (2.0)

Each row specifies its threshold. Proportions are n/N (%) with Wilson 95% CIs. IQR = Q3 − Q1. Lower values indicate more frequent experiences, except that item 17 ranges from a very negative to a very positive perceived effect. Item 20 has one missing response. Item 19 explicitly attributes thoughts of transfer to fatigue; items 20 and 21 do not. For item 16, including sometimes gives 46/145 (31.7%).

**Table 5:** Exploratory logistic regression for fatigue-attributed thoughts of leaving at least sometimes.

Predictor	Adjusted OR (95% CI)	*p*
Rotating work vs. either daytime schedule	2.16 (0.997–4.682)	0.051
Female vs. male	1.28 (0.40–4.13)	0.675
Experience, per year	1.02 (0.99–1.05)	0.249
Bachelor’s vs. secondary nursing education	1.01 (0.47–2.14)	0.984
Master’s vs. secondary nursing education	0.73 (0.20–2.71)	0.641

Outcome: item 8 codes 1–3 (always, often or sometimes) versus codes 4–5 (rarely or never). Model *n* = 142, with 76 events and 66 non-events. The full-sample proportion is 76/145 (52.4%); the model proportion is 76/142 (53.5%). All covariates were entered simultaneously. Reference groups: either daytime schedule, male sex and secondary nursing education. Experience is continuous per year. Age was excluded because r = 0.973 with experience. The CI for rotating work is given to three decimals to show that it includes 1. OR, odds ratio; CI, confidence interval.

## Data Availability

The raw data supporting the conclusions of this article will be made available by the authors on request.

## Supplementary Materials

The following supporting information can be downloaded at: https://www.mdpi.com/article/10.3390/healthcare14203363/s1, Supplementary File S1: Submitted English questionnaire and informed consent with a Croatian translation; Supplementary File S2: STROBE checklist; Supplementary File S3: Response distributions and additional statistical analyses.

## Appendix A

The analysis comprises 21 variables covering demographics, work schedules and individual occupational experiences. Supplementary File S1 contains the submitted English questionnaire and a Croatian version, with all response options; Supplementary File S3 provides the response distributions.

Part 1—Sociodemographic and work-related variables: age, sex, years of healthcare work experience, education and work schedule. For regression, education was coded as secondary nursing education, bachelor’s degree and master’s degree; the postgraduate category had no observations. Schedules in the analytical dataset were rotating 12/24/12/48, morning only and combined morning/afternoon.

Part 2—Fatigue and work experiences: (6) frequency of fatigue at work; (7) perceived effect of fatigue on ability to work; (8) thoughts of leaving the current job because of fatigue; (9) attribution of fatigue to long shifts; (10) schedule satisfaction; (11) attribution of fatigue to insufficient breaks; (12) physical exhaustion; and (13) emotional exhaustion because of work.

Part 3—Perceived consequences and thoughts of leaving: (14) perceived effect on patient safety; (15) perceived effect on decision-making; (16) errors attributed to fatigue; (17) perceived effect on communication; (18) perceived reduction in productivity; (19) thoughts of transfer within the institution because of fatigue; (20) thoughts of moving to another institution; and (21) thoughts of leaving nursing. Items 20 and 21 do not attribute thoughts to fatigue.
