# Supplementary material for: Self-Reported Fatigue and Fatigue-Attributed Thoughts of Leaving the Current Job Among Internal Medicine Nurses: An Exploratory Cross-Sectional Study
Source: Healthcare (Basel). 2026 Oct 9;14(20):3363. doi: 10.3390/healthcare14203363 (PMC13704089; doi:10.3390/healthcare14203363)
Supplement: Supplementary file 1 [file healthcare-14-03363-s001.zip › Supplementary File_S1_Questionnaire_Bilingual.pdf]

Supplementary File S1

Questionnaire and informed consent
Croatian and English versions
Manuscript: healthcare-4564019

This supplement presents the questionnaire and informed-consent text in Croatian and English, including all 21 questions and response options.

A Hrvatska verzija

Umor među medicinskim sestrama: uzroci, posljedice i utjecaj na fluktuaciju radne snage

INFORMIRANI PRISTANAK SUDIONIKA

Cilj ovog istraživanja jest istražiti uzroke i posljedice umora te njegov utjecaj na fluktuaciju radne snage među medicinskim sestrama.

Molimo Vas da ispunite upitnik o svojim iskustvima s umorom na radnom mjestu, uključujući njegovu učestalost, uzroke i posljedice za Vaš radni život. Ispunjavanje upitnika traje približno 5–10 minuta.

Sudjelovanje u ovom istraživanju je dobrovoljno. Možete odlučiti ne sudjelovati ili u bilo kojem trenutku odustati od sudjelovanja bez ikakvih negativnih posljedica.

Vaši će odgovori ostati strogo povjerljivi. Podatci će se obrađivati anonimno, bez mogućnosti identifikacije pojedinih sudionika. Informacije će se koristiti isključivo za potrebe ovog istraživanja.

Nema poznatih rizika povezanih s Vašim sudjelovanjem u ovom istraživanju. Moguće koristi uključuju bolje razumijevanje umora među medicinskim sestrama i poboljšanje pristupa njegovu rješavanju.

Za sva pitanja o istraživanju možete se obratiti članici istraživačkog tima Mileni Gabrić putem e-pošte: milena.gabric@kbcsm.hr.

Potpisivanjem ovog dokumenta izjavljujete da ste pročitali informacije o istraživanju i da ste informirani o svojem sudjelovanju. Također potvrđujete da u istraživanju sudjelujete dobrovoljno.

Potpis sudionika: ________________________   Datum: _______________

UPITNIK

Dio 1 Sociodemografski podatci

1. Dob (godine):

____

2. Spol:

☐ Muško ☐ Žensko ☐ Ne želim se izjasniti

3. Godine radnog iskustva u zdravstvu:

____

4. Najviša završena razina obrazovanja:

☐ Srednja škola (sestrinstvo) ☐ Prijediplomski studij sestrinstva ☐ Diplomski studij

5. Trenutačni raspored rada:

☐ Smjenski rad (12/24/12/48) ☐ Isključivo jutarnje smjene ☐ Kombinirane jutarnje i poslijepodnevne smjene

Dio 2 Učestalost i samoprocjena umora

6. Koliko često osjećate umor na poslu?

☐ Svaki dan ☐ Nekoliko puta tjedno ☐ Jednom tjedno ☐ Rijetko ☐ Nikada

7. Koliko često smatrate da umor utječe na Vašu sposobnost obavljanja posla?

☐ Uvijek ☐ Često ☐ Ponekad ☐ Rijetko ☐ Nikada

8. Koliko često razmišljate o napuštanju trenutačnog radnog mjesta zbog umora?

☐ Uvijek ☐ Često ☐ Ponekad ☐ Rijetko ☐ Nikada

9. Koliko često osjećate da je Vaš umor posljedica dugih smjena?

☐ Uvijek ☐ Često ☐ Ponekad ☐ Rijetko ☐ Nikada

10. Koliko ste zadovoljni trenutačnim rasporedom smjena?

☐ Vrlo zadovoljan/na ☐ Zadovoljan/na ☐ Ni zadovoljan/na ni nezadovoljan/na ☐ Nezadovoljan/na ☐ Vrlo nezadovoljan/na

11. Koliko često smatrate da nedostatak stanki tijekom smjene doprinosi Vašem umoru?

☐ Uvijek ☐ Često ☐ Ponekad ☐ Rijetko ☐ Nikada

12. Koliko često osjećate fizičku iscrpljenost na poslu?

☐ Svaki dan ☐ Nekoliko puta tjedno ☐ Jednom tjedno ☐ Rijetko ☐ Nikada

13. Koliko često osjećate emocionalnu iscrpljenost zbog posla?

☐ Svaki dan ☐ Nekoliko puta tjedno ☐ Jednom tjedno ☐ Rijetko ☐ Nikada

Dio 3 Posljedice umora

14. Koliko često smatrate da umor utječe na sigurnost pacijenata?

☐ Uvijek ☐ Često ☐ Ponekad ☐ Rijetko ☐ Nikada

15. Koliko često osjećate da umor utječe na Vaše donošenje odluka?

☐ Uvijek ☐ Često ☐ Ponekad ☐ Rijetko ☐ Nikada

16. Koliko često dolazi do pogrešaka u radu koje pripisujete umoru?

☐ Uvijek ☐ Često ☐ Ponekad ☐ Rijetko ☐ Nikada

17. Kako umor utječe na Vašu komunikaciju s kolegama i pacijentima?

☐ Vrlo negativno ☐ Negativno ☐ Neutralno ☐ Pozitivno ☐ Vrlo pozitivno

18. Koliko često smatrate da umor smanjuje Vašu produktivnost na poslu?

☐ Uvijek ☐ Često ☐ Ponekad ☐ Rijetko ☐ Nikada

19. Koliko često razmišljate o prelasku na drugo radno mjesto unutar iste ustanove zbog umora?

☐ Uvijek ☐ Često ☐ Ponekad ☐ Rijetko ☐ Nikada

20. Koliko često razmišljate o prelasku u drugu zdravstvenu ustanovu?

☐ Uvijek ☐ Često ☐ Ponekad ☐ Rijetko ☐ Nikada

21. Koliko često razmišljate o napuštanju struke?

☐ Uvijek ☐ Često ☐ Ponekad ☐ Rijetko ☐ Nikada

B English version

Fatigue Among Nurses: Causes, Consequences, and Impact on Workforce Turnover

PARTICIPANT INFORMED CONSENT

The aim of this study is to investigate the causes and consequences of fatigue and its impact on workforce turnover among nurses.

Please complete the survey concerning your experiences of fatigue in the workplace, including its frequency, causes, and the consequences it has on your working life. Completing the survey will take approximately 5–10 minutes.

Participation in this study is voluntary. You may decide not to participate, or withdraw from participation at any time, without any negative consequences.

Your responses will remain strictly confidential. The data will be processed anonymously, without any possibility of identifying individual participants. The information will be used exclusively for the purposes of this study.

There are no known risks associated with your participation in this study. The benefits include a potentially improved understanding of, and approach to addressing, fatigue among nurses.

For any questions regarding the study, you may contact the research team member, Milena Gabrić, by e-mail: milena.gabric@kbcsm.hr.

By signing this document, you declare that you have read the information about the study and that you have been informed about your participation. You also confirm that you are taking part in the study voluntarily.

Participant's signature: ________________________ Date: _______________

SURVEY

Part 1 Sociodemographic Data

1. Age (years):

____

2. Sex:

☐ Male ☐ Female ☐ Prefer not to say

3. Years of work experience in healthcare:

____

4. Highest completed level of education:

☐ Secondary school (nursing) ☐ Bachelor of nursing (undergraduate) ☐ Master's degree (graduate)

5. Current work schedule:

☐ Shift work (12/24/12/48) ☐ Morning shifts only ☐ Combined morning and afternoon shifts

Part 2 Frequency and Self-Assessment of Fatigue

6. How often do you feel tired at work?

☐ Every day ☐ Several times per week ☐ Once per week ☐ Rarely ☐ Never

7. How often do you think fatigue affects your ability to do your job?

☐ Always ☐ Often ☐ Sometimes ☐ Rarely ☐ Never

8. How often do you consider leaving your current job because of fatigue?

☐ Always ☐ Often ☐ Sometimes ☐ Rarely ☐ Never

9. How often do you feel your fatigue is a result of long shifts?

☐ Always ☐ Often ☐ Sometimes ☐ Rarely ☐ Never

10. How satisfied are you with your current shift schedule?

☐ Very satisfied ☐ Satisfied ☐ Neutral ☐ Dissatisfied ☐ Very dissatisfied

11. How often do you think the lack of breaks during a shift contributes to your fatigue?

☐ Always ☐ Often ☐ Sometimes ☐ Rarely ☐ Never

12. How often do you feel physical exhaustion at work?

☐ Every day ☐ Several times per week ☐ Once per week ☐ Rarely ☐ Never

13. How often do you feel emotional exhaustion because of work?

☐ Every day ☐ Several times per week ☐ Once per week ☐ Rarely ☐ Never

Part 3 Consequences of Fatigue

14. How often do you believe fatigue affects patient safety?

☐ Always ☐ Often ☐ Sometimes ☐ Rarely ☐ Never

15. How often do you feel fatigue affects your decision-making?

☐ Always ☐ Often ☐ Sometimes ☐ Rarely ☐ Never

16. How often do work errors occur that you attribute to fatigue?

☐ Always ☐ Often ☐ Sometimes ☐ Rarely ☐ Never

17. How does fatigue affect your communication with colleagues and patients?

☐ Very negative ☐ Negative ☐ Neutral ☐ Positive ☐ Very positive

18. How often do you think fatigue reduces your productivity at work?

☐ Always ☐ Often ☐ Sometimes ☐ Rarely ☐ Never

19. How often do you consider transferring to another position within the same institution because of fatigue?

☐ Always ☐ Often ☐ Sometimes ☐ Rarely ☐ Never

20. How often do you consider moving to another healthcare institution?

☐ Always ☐ Often ☐ Sometimes ☐ Rarely ☐ Never

21. How often do you consider leaving the profession?

☐ Always ☐ Often ☐ Sometimes ☐ Rarely ☐ Never
