# Supplementary material for: Self-Reported Fatigue and Fatigue-Attributed Thoughts of Leaving the Current Job Among Internal Medicine Nurses: An Exploratory Cross-Sectional Study
Source: Healthcare (Basel). 2026 Oct 9;14(20):3363. doi: 10.3390/healthcare14203363 (PMC13704089; doi:10.3390/healthcare14203363)
Supplement: Supplementary file 1 [file healthcare-14-03363-s001.zip › Supplementary File_S2_STROBE_Checklist.pdf]

Supplementary File S2

STROBE checklist for the revised cross-sectional study
Manuscript: healthcare-4564019

Locations refer to the revised manuscript. The study is reported as exploratory; no claim of prospectively registered hypotheses or analytical cut-offs is made.

Item	Topic	STROBE recommendation	Revised location / explanation
1 (a)	Title and abstract	Indicate the study's design with a commonly used term in the title or the abstract	Title and Abstract (p. 1); Title and Abstract (p. 1, lines 14–33)
1 (b)	Title and abstract	Provide in the abstract an informative and balanced summary of what was done and what was found	Title and Abstract (p. 1, lines 14–33)
2	Background/rationale	Explain the scientific background and rationale for the investigation being reported	Introduction; 1. Introduction (p. 2, lines 72–78)
3	Objectives	State specific objectives, including any prespecified hypotheses	1. Introduction (pp. 2–3, lines 86–97)
4	Study design	Present key elements of study design early in the paper	2.1. Study Design and Setting (p. 3, lines 100–110)
5	Setting	Describe the setting, locations, and relevant dates, including periods of recruitment, exposure, and data collection	2.1. Study Design and Setting (p. 3, lines 100–110)
6 (a)	Participants	Give the eligibility criteria, and the sources and methods of selection of participants	2.2. Participants and Sample Size (p. 3, lines 112–118); 2.2. Participants and Sample Size (p. 3, lines 119–126)
7	Variables	Clearly define all outcomes, exposures, predictors, potential confounders, and effect modifiers	Sections 2.3–2.4; Appendix A; Supplementary S1
8	Data sources/measurement	For each variable of interest, give sources of data and details of methods of assessment (measurement)	Section 2.3; Appendix A; Supplementary S1
9	Bias	Describe any efforts to address potential sources of bias	2.2. Participants and Sample Size (p. 3, lines 119–126); Section 4.5
10	Study size	Explain how the study size was arrived at	2.2. Participants and Sample Size (p. 3, lines 112–118)
11	Quantitative variables	Explain how quantitative variables were handled; if applicable, describe which groupings were chosen and why	2.4. Statistical Analysis (p. 4, lines 174–185); 2.4. Statistical Analysis (pp. 4–5, lines 186–198)

Item	Topic	STROBE recommendation	Revised location / explanation
12 (a)	Statistical methods	Describe all statistical methods, including those used to control for confounding	Section 2.4; Supplementary S3.5
12 (b)	Statistical methods	Describe any methods used to examine subgroups and interactions	Sections 2.4, 3.3 and 3.5; Supplementary S3.3–S3.5. No interaction analysis.
12 (c)	Statistical methods	Explain how missing data were addressed	2.4. Statistical Analysis (p. 4, lines 168–173); 2.4. Statistical Analysis (p. 5, lines 199–208)
12 (d)	Statistical methods	If applicable, describe analytical methods taking account of sampling strategy	No sampling weights used; census of eligible nurses attempted (Section 2.2).
12 (e)	Statistical methods	Describe any sensitivity analyses	2.4. Statistical Analysis (p. 5, lines 209–221); 3.5. Exploratory Multivariable Associations (pp. 8–9, lines 311–319); Supplementary S3
13 (a)	Participants	Report the numbers of individuals at each stage of the study	2.2. Participants and Sample Size (p. 3, lines 112–118); Table 1
13 (b)	Participants	Give reasons for non-participation at each stage	2.2. Participants and Sample Size (p. 3, lines 112–118); 2.2. Participants and Sample Size (p. 3, lines 119–126). Reasons among eligible non-participants were not recorded.
13 (c)	Participants	Consider use of a flow diagram	No diagram; recruitment numbers are stated in Section 2.2.
14 (a)	Descriptive data	Give characteristics of study participants and information on exposures and potential confounders	Section 3.1; Table 1
14 (b)	Descriptive data	Indicate the number of participants with missing data for each variable of interest	Sections 2.4 and 3.1; Tables 1 and 4; Supplementary S3.1
15	Outcome data	Report numbers of outcome events or summary measures	Sections 3.2–3.5; Tables 2–5; Supplementary S3
16 (a)	Main results	Give unadjusted estimates and, if applicable, confounder-adjusted estimates and their precision (e.g., 95% CI)	Sections 3.2–3.5; Tables 2–5; Supplementary S3
16 (b)	Main results	Report category boundaries when continuous variables were categorized	Section 2.4; Table footnotes; Supplementary S3.2–S3.4
16 (c)	Main results	If relevant, consider translating estimates of relative risk into absolute risk	Not applicable: exploratory cross-sectional associations; no projected risk estimates.

Item	Topic	STROBE recommendation	Revised location / explanation
17	Other analyses	Report other analyses done—e.g., subgroups, interactions, and sensitivity analyses	Sections 3.3 and 3.5; Supplementary S3
18	Key results	Summarise key results with reference to study objectives	4. Discussion (p. 9, lines 328–332)
19	Limitations	Discuss limitations, taking into account sources of potential bias or imprecision	Section 4.5
20	Interpretation	Give a cautious overall interpretation of results considering objectives, limitations, and other evidence	Sections 4.1–4.4 and 5
21	Generalisability	Discuss the generalisability (external validity) of the study results	4.5. Limitations (p. 10, lines 388–396)
22	Funding	Give the source of funding and the role of the funders for the present study	Funding statement: no external funding.
