# Supplementary material for: Self-Reported Fatigue and Fatigue-Attributed Thoughts of Leaving the Current Job Among Internal Medicine Nurses: An Exploratory Cross-Sectional Study
Source: Healthcare (Basel). 2026 Oct 9;14(20):3363. doi: 10.3390/healthcare14203363 (PMC13704089; doi:10.3390/healthcare14203363)
Supplement: Supplementary file 1 [file healthcare-14-03363-s001.zip › Supplementary File_S3_Additional_Analyses.pdf]

Supplementary File S3

Response distributions and additional statistical analyses
Manuscript: healthcare-4564019

All results use the supplied analytical dataset of 145 respondents. Items were analysed individually, without composite scores. The analyses below supplement Sections 2.4 and 3 of the revised manuscript. Sensitivity analyses were conducted during revision and are exploratory.

Age and experience are represented as exact years in the analytical dataset. Work schedule is represented by three categories. Numerical codes and labels follow the dataset coding instructions and the submitted English questionnaire; code 1 for the relevant frequency items is labelled “always” (“uvijek”).

S3.1. Missing responses and denominators

Variable	Valid n	Missing / excluded	Additional detail
Age	145	0	Exact years in dataset
Sex	144	1	One additional respondent preferred not to disclose
Experience	144	1	Exact years in dataset
Education and schedule	145	0	Complete
Item 20	144	1	Other items 6–21 complete
Main regression	142	3	Missing sex, undisclosed sex, or missing experience

S3.2. Five-category distributions

Cells show n (% of available responses). For items 6, 12 and 13: 1 = every day; 2 = several times per week; 3 = once per week; 4 = rarely; 5 = never. For other frequency items: 1 = always; 2 = often; 3 = sometimes; 4 = rarely; 5 = never. Item 10: very satisfied to very dissatisfied. Item 17: very negative to very positive.

Item	N	1	2	3	4	5
6. Fatigue at work	145	35 (24.1)	68 (46.9)	26 (17.9)	15 (10.3)	1 (0.7)
7. Ability to work	145	13 (9.0)	45 (31.0)	53 (36.6)	30 (20.7)	4 (2.8)

Item	N	1	2	3	4	5
8. Leaving because of fatigue	145	4 (2.8)	25 (17.2)	47 (32.4)	36 (24.8)	33 (22.8)
9. Long shifts	145	20 (13.8)	35 (24.1)	49 (33.8)	30 (20.7)	11 (7.6)
10. Schedule satisfaction	145	29 (20.0)	77 (53.1)	34 (23.4)	3 (2.1)	2 (1.4)
11. Insufficient breaks	145	20 (13.8)	43 (29.7)	52 (35.9)	19 (13.1)	11 (7.6)
12. Physical exhaustion	145	22 (15.2)	62 (42.8)	28 (19.3)	31 (21.4)	2 (1.4)
13. Emotional exhaustion	145	27 (18.6)	59 (40.7)	23 (15.9)	30 (20.7)	6 (4.1)
14. Patient safety	145	14 (9.7)	23 (15.9)	50 (34.5)	32 (22.1)	26 (17.9)
15. Decision-making	145	10 (6.9)	18 (12.4)	45 (31.0)	51 (35.2)	21 (14.5)
16. Self-attributed errors	145	2 (1.4)	6 (4.1)	38 (26.2)	68 (46.9)	31 (21.4)
17. Communication	145	3 (2.1)	41 (28.3)	96 (66.2)	3 (2.1)	2 (1.4)
18. Productivity	145	7 (4.8)	22 (15.2)	73 (50.3)	34 (23.4)	9 (6.2)
19. Internal transfer	145	1 (0.7)	22 (15.2)	31 (21.4)	35 (24.1)	56 (38.6)
20. Other institution	144	3 (2.1)	14 (9.7)	42 (29.2)	23 (16.0)	62 (43.1)
21. Leaving nursing	145	8 (5.5)	25 (17.2)	30 (20.7)	25 (17.2)	57 (39.3)

Item 8: codes 1–3 give 76/145 (52.4%); codes 1–2 give 29/145 (20.0%). These are distinct descriptive definitions, not validated clinical cut-offs. Item 19 explicitly refers to fatigue; items 20 and 21 do not.

S3.3. Categorical subgroup comparisons

Fatigue at least weekly (item 6, codes 1–3)

Group	Yes, n/N (%)	No, n/N (%)	Exact p	V
Secondary nursing education	60/70 (85.7)	10/70 (14.3)	0.214	0.13
Bachelor's degree	59/63 (93.7)	4/63 (6.3)	—	—
Master's degree	10/12 (83.3)	2/12 (16.7)	—	—
Rotating 12/24/12/48	76/81 (93.8)	5/81 (6.2)	0.012	0.26
Morning only	49/57 (86.0)	8/57 (14.0)	—	—
Morning/afternoon	4/7 (57.1)	3/7 (42.9)	—	—

Thoughts of leaving at least sometimes (item 8, codes 1–3)

Group	Yes, n/N (%)	No, n/N (%)	Exact p	V
Secondary nursing education	39/70 (55.7)	31/70 (44.3)	0.655	0.08
Bachelor's degree	32/63 (50.8)	31/63 (49.2)	—	—
Master's degree	5/12 (41.7)	7/12 (58.3)	—	—
Rotating 12/24/12/48	48/81 (59.3)	33/81 (40.7)	0.143	0.17
Morning only	24/57 (42.1)	33/57 (57.9)	—	—
Morning/afternoon	4/7 (57.1)	3/7 (42.9)	—	—

p-values compare the three education or schedule groups using the two-sided Fisher–Freeman–Halton exact test; V is Cramér's V. Dashes indicate the same group-level test. These exploratory tests were not adjusted for multiplicity. With both daytime schedules combined, weekly fatigue was reported by 76/81 rotating and 53/64 daytime nurses (Fisher's exact p = 0.059; unadjusted OR 3.15).

S3.4. Experience-group comparisons

Item	<15 years n = 68 Median	≥15 years n = 76 Median	p	|r|
8. Leaving because of fatigue	3.5	3.0	0.737	0.03

Item	<15 years<br>n = 68<br>Median	≥15 years<br>n = 76<br>Median	p	|r|
10. Schedule satisfaction	2.0	2.0	0.326	0.09
11. Insufficient breaks	3.0	3.0	0.451	0.07
6. Fatigue at work	2.0	2.0	0.650	0.04

One experience value is missing. Two-sided Mann–Whitney U tests with tie and continuity corrections; unadjusted exploratory p-values. |r| is the absolute rank-biserial correlation. The 15-year division is descriptive and not a validated threshold.

S3.5. Logistic regression and sensitivity analyses

The main outcome is item 8 codes 1–3 versus 4–5. Predictors were entered simultaneously. Reference categories are either daytime schedule, male sex and secondary nursing education. Experience is entered per year. Age is omitted because its correlation with experience is 0.973. Estimates use maximum likelihood and Wald 95% CIs. The main sample contains 76 events and 66 non-events; all three excluded respondents were non-events.

Model A. Main analysis

n = 142; events = 76; non-events = 66. The model converged. Likelihood-ratio $\chi^2(5)$ = 5.38, p = 0.371; AIC = 202.77; Nagelkerke R^2 = 0.050; Brier score = 0.240.

Term	β	SE	OR (95% CI)	p
Intercept	-0.804	0.735	0.45 (0.11–1.89)	0.274
Rotating vs daytime work	0.770	0.395	2.16 (0.997–4.682)	0.051
Experience, per year	0.017	0.015	1.02 (0.99–1.05)	0.249
Bachelor's vs secondary	0.008	0.384	1.01 (0.47–2.14)	0.984
Master's vs secondary	-0.311	0.667	0.73 (0.20–2.71)	0.641
Female vs male	0.250	0.596	1.28 (0.40–4.13)	0.675

Model B. Stricter outcome: always/often versus sometimes/rarely/never

n = 142; events = 29; non-events = 113. The model converged. Likelihood-ratio $\chi^2(5)$ = 1.44, p = 0.920; AIC = 154.32; Nagelkerke R^2 = 0.016; Brier score = 0.161.

Term	β	SE	OR (95% CI)	p
Intercept	-2.260	0.974	0.10 (0.02–0.70)	0.020
Rotating vs daytime work	0.438	0.479	1.55 (0.61–3.96)	0.360
Experience, per year	0.005	0.017	1.01 (0.97–1.04)	0.757
Bachelor's vs secondary	0.021	0.474	1.02 (0.40–2.59)	0.964
Master's vs secondary	0.407	0.765	1.50 (0.34–6.73)	0.595
Female vs male	0.544	0.816	1.72 (0.35–8.53)	0.505

This model has only 29 events for five predictor coefficients. Its estimates are particularly imprecise and are used only to examine threshold sensitivity.

Model C. Sex omitted; original complete-case sample

n = 142; events = 76; non-events = 66. The model converged. Likelihood-ratio $\chi^2(4)$ = 5.20, p = 0.267; AIC = 200.95; Nagelkerke R^2 = 0.048; Brier score = 0.240.

Term	β	SE	OR (95% CI)	p
Intercept	-0.596	0.542	0.55 (0.19–1.59)	0.271
Rotating vs daytime work	0.753	0.392	2.12 (0.98–4.58)	0.055
Experience, per year	0.018	0.014	1.02 (0.99–1.05)	0.211
Bachelor's vs secondary	0.021	0.382	1.02 (0.48–2.16)	0.956
Master's vs secondary	-0.301	0.665	0.74 (0.20–2.73)	0.651

Model D. Sex omitted; missing/undisclosed sex retained

n = 144; events = 76; non-events = 68. The model converged. Likelihood-ratio $\chi^2(4)$ = 5.72, p = 0.221; AIC = 203.46; Nagelkerke R^2 = 0.052; Brier score = 0.240.

Term	β	SE	OR (95% CI)	p
Intercept	-0.749	0.531	0.47 (0.17–1.34)	0.158
Rotating vs daytime work	0.800	0.389	2.23 (1.04–4.77)	0.040
Experience, per year	0.021	0.014	1.02 (0.99–1.05)	0.139
Bachelor's vs secondary	0.104	0.378	1.11 (0.53–2.32)	0.784

Term	β	SE	OR (95% CI)	p
Master’s vs secondary	-0.216	0.664	0.81 (0.22–2.96)	0.744

Model E. Quadratic experience term added

n = 142; events = 76; non-events = 66. The model converged. Likelihood-ratio $\chi^2(6)$ = 9.27, p = 0.159; AIC = 200.88; Nagelkerke R^2 = 0.084; Brier score = 0.233.

Term	β	SE	OR (95% CI)	p
Intercept	-0.510	0.764	0.60 (0.13–2.69)	0.505
Rotating vs daytime work	0.854	0.403	2.35 (1.07–5.18)	0.034
Experience: linear term	0.027	0.016	1.03 (1.00–1.06)	0.084
Bachelor's vs secondary	-0.073	0.391	0.93 (0.43–2.00)	0.852
Master's vs secondary	-0.456	0.680	0.63 (0.17–2.41)	0.503
Female vs male	0.216	0.612	1.24 (0.37–4.12)	0.724
Scaled centred experience^2	-0.243	0.125	0.78 (0.61–1.00)	0.052

The added term is $[(\text{experience} - 18.570423)/10]^2$, while the linear term remains in years. Its OR describes one unit of this transformed term and is not an OR per year. The linear and quadratic terms must be interpreted jointly; neither coefficient alone describes a constant annual effect. The likelihood-ratio comparison with Model A gives $\chi^2(1)$ = 3.888, p = 0.049. This is an exploratory functional-form check.

Main-model VIFs range from 1.08 to 1.30. The estimates are similar in direction, but statistical significance changes with outcome definition, case inclusion and the functional form of experience. No sensitivity model was selected to replace the main result because it produced a smaller p-value. These analyses do not establish a robust schedule association or a causal effect.
